# Impact of anastomotic leakage on long-term oncologic outcome and its related factors in rectal cancer

**DOI:** 10.1097/MD.0000000000004367

**Published:** 2016-07-29

**Authors:** Gyoung Tae Noh, Yeo Shen Ann, Chinock Cheong, Jeonghee Han, Min Soo Cho, Hyuk Hur, Byung Soh Min, Kang Young Lee, Nam Kyu Kim

**Affiliations:** Department of Surgery, Severance Hospital, Yonsei University College of Medicine, Seoul, Korea.

**Keywords:** anastomotic leakage, oncologic outcome, prognosis related factors, rectal cancer

## Abstract

Anastomotic leakage (AL) is a well-known cause of morbidity after low anterior resection (LAR) for rectal cancer, but its impact on oncologic outcome is not well understood. The aim of this study is to investigate the impact of AL on long-term oncologic outcome and to identify factors associated with AL that may affect prognosis after LAR for rectal cancer.

A retrospective analysis of patients who underwent curative resection for rectal cancer without diverting stoma was performed. To investigate AL related factors that may be associated with oncologic outcome, Clavien-Dindo grades, prognostic nutritional indices (PNI) and inflammatory indices were included.

One hundred and one patients out of a total of 1258 patients developed postoperative AL, giving an AL rate of 8.0%. Patients with AL showed poorer disease-free survival (DFS), than patients without AL (hazard ratio [HR] = 1.6; 95% confidence intervals [CI]: 1.1–2.5; *P* = 0.01). In patients who developed AL, age over 60 (HR = 2.2; 95% CI: 1.1–4.7; *P* = 0.033), advanced pathologic stage (HR = 2.4; 95% CI: 1.4–4.0; *P* = 0.001), suppressed neutrophil-proportion (≤80%) (HR = 2.6; 95% CI: 1.2–5.8; *P* = 0.019) and PNI <36 (HR = 3.5; 95% CI: 1.2–9.6; *P* = 0.018) were associated with poorer DFS.

AL was associated with poorer DFS. In patients with AL, a suppressed neutrophil-proportion and decreased PNI below 36 were associated with tumor recurrence.

## 1 Introduction

Anastomotic leakage (AL) is one of the major causes of morbidity after low anterior resection (LAR) for rectal cancer, with reported incidences ranging from 3.9% to 19.2%.^[1–6]^ Introduction of stapling anastomosis along with advancements in surgical techniques have resulted in an increased number of sphincter preserving rectal resections, however this has consequently increased the likelihood of AL.^[7–9]^

It is evident that AL leads to short term morbidity, mortality, and poor functional results.^[10]^ There have been many efforts to prevent AL by identifying high-risk patients. According to recent studies, male gender, preoperative chemo-radiotherapy, lower tumor location as well as increased number of stapler application for distal rectal transection have been established risk factors for AL.^[11–13]^ Although there seems to be an overall consensus that AL is related to poor prognosis, the oncologic consequence in patients with AL is uncertain. There are several contradictory studies with respect to long-term and cancer-specific survival of patients with AL. In a large-scale population-based cohort study, AL was not associated with an increased local recurrence rate.^[14]^ A recent retrospective study using propensity score analysis also reported that AL has no impact on long-term survival in both overall and cancer-specific survival.^[15]^ In contrast to these reports, a multicenter study found an impaired overall survival (OS) after AL, but there was no association with poorer cancer-specific survival.^[16]^ Another multicenter study analyzing the impact of AL after total mesorectal excision for rectal cancer demonstrated that AL resulted in inferior oncologic outcomes with respect to both disease-free survival (DFS) and OS.^[17]^ Similarly, a recent meta-analysis of 21 trials concluded that AL has a negative prognostic impact on local recurrence and cancer-specific survival.^[18]^ The discrepancy between previous reports may reflect the heterogeneous nature of the prognosis after AL. In this context, clarifying factors that affect the prognosis after AL may have value to define subgroups of different prognosis, which will in turn lead to a more tailored treatment strategy for individual patients.

We have previously demonstrated that the clinical features of AL are different according to whether open or minimal invasive surgery is performed amongst other risk factors.^[11]^ This reflects that variable physiologic and biochemical processes after different surgical approaches may influence the clinical manifestation of AL. In this context, we hypothesize that the oncologic outcomes after AL may differ in relation to various clinical, biochemical, and pathological factors. To investigate this issue, we reviewed our patients’ characteristics and compared the oncologic outcome between patients with and without AL. We further analyzed the data to characterize in detail the factors associated with poor outcomes in patients with AL.

## 2 Materials and methods

From January 2006 to December 2012, the medical records of consecutive patients who underwent an elective operation of LAR with colorectal anastomosis using the double-stapling method for the treatment of rectal cancer at a high volume tertiary medical institution were reviewed retrospectively. All patients with a pathological diagnosis of rectal adenocarcinoma located within 15 cm from anal verge were included. Of 1961 patients diagnosed as rectal cancer and performed surgery under curative intention, we excluded 426 patients who underwent surgical procedures other than LAR with double stapling anastomosis such as local excision, Hartmann's operation, hand-sewn coloanal anastomosis and abdominoperineal resection, as well as 278 patients who had a diverting stoma. We defined AL as the breakdown of a colorectal anastomosis with an infected fluid collection in the pelvic cavity within 60 days after the index operation. AL was diagnosed by computed tomography or by clinical symptoms and signs including change of drain color and/or fever with peritonitis. As proximal diversion may prevent the clinical manifestations of AL, patients who underwent protective stoma formation were not included in this study. The variables included in the analysis comparing patients with and without AL were as follows: age, gender, American society of anesthesiologists (ASA) physical status grade, preoperative carcinoembryonic antigen (CEA) level, surgical approach (open surgery or minimal invasive surgery), pathologic stage, histologic grade, lymphovascular invasion, local recurrence, pre- or postoperative chemo-radiotherapy, interval from surgery to initiation of postoperative adjuvant treatment, duration of hospital stay after surgery, duration of follow-up, DFS and OS. Minimal invasive surgery included both laparoscopic and robotic surgery. Pathologic staging was based on the 7th edition of the American Joint Commission on Cancer tumor-node-metastasis (TNM) system.^[19]^ Follow-up strategies for the patients with and without AL were identical. Patients were followed up every 6 months for the first 3 years after surgery and yearly thereafter. Each follow-up visit included a medical history, a physical examination, and measurement of the serum CEA concentration. Routine imaging studies consist of chest radiography and computed tomography on chest, abdomen, and pelvis. Chest radiography and abdominopelvic CT were performed 6 months after surgery for 3 years and annually thereafter. Colonoscopy was performed annually after surgery. Ultrasonography, whole-body bone scintigraphy and positron emission tomography (PET) were performed when there was suspicion of recurrence on routine imaging studies. Patients who experienced AL underwent same follow-up schedule as patients without AL after their treatment for AL was over. Local recurrence was defined as any recurrent tumor growth within the pelvic cavity or perineal area confirmed by clinical, radiological, or pathologic evaluation, and the other tumor recurrence events were categorized as systemic recurrences. DFS was defined as months from the date of surgery to the date of detection of recurrence, last follow-up or death. OS was defined as months from the date of surgery to the date of death or last follow-up. Among these variables, age, gender, ASA grade, surgical method, CEA, pathologic stage, histologic grade, and lymphovascular invasion were included in the survival analysis. Furthermore, we added factors related to perioperative treatment modalities (pre- or postoperative chemo-radiotherapy) into the analysis to adjust for their effect on oncologic outcomes. In the patients who required adjuvant treatment, postoperative chemo-radiotherapy was performed by clinical oncologists and the timing of initiation was determined considering patients’ recovery after surgery. Patients received identical adjuvant chemotherapeutic regimen regardless of the experience of AL. The interval between the index operation and the initiation of postoperative adjuvant treatment was categorized and analyzed to adjust for confounding. To investigate the factors affecting oncologic outcomes in patients with AL, we analyzed variables such as age, gender, ASA grade, surgical method, CEA, pathologic stage, histologic grade, and lymphovascular invasion. Additionally, the severity of the AL according to the Clavien-Dindo classification (grade I, deviation from the normal postoperative course without the need for therapy; grade II, complications requiring pharmacological treatment; grade III, complications requiring surgical, endoscopic or radiological intervention; grade IV, life-threatening complications requiring intensive care; and grade V, death), leukocyte count, neutrophil-proportion (out of total leukocytes) and prognostic nutritional index (PNI) were included as factors assessing the clinical severity and degree of inflammatory or immunologic reaction.^[20]^ All of these biochemical indices were assessed at the time of the diagnosis of AL. The PNI was calculated according to the following formula: 10 × albumin (g/dl)  + 0.005*x* total lymphocyte count (per mm^3^).^[21]^ This study was approved by the Institutional Review Board of Severance Hospital (4-2016-0153).

### 2.1 Statistical analysis

All statistical analyses were performed using SPSS Statistics (version 20.0., IBM Corp., Armonk, NY), with the exception of calculating cut-off value of PNI. Categorical variables were analyzed using the *χ*^2^ test and continuous variables were analyzed using the Student *t* test. Differences in survival between groups with and without AL were compared using the Kaplan–Meier method and tested with the log-rank test. Factors associated with DFS and OS were analyzed by a Cox-proportional hazards regression model. For the Cox-proportional hazards regression, continuous variables were dichotomized according to the clinical implications or using the mean value of each variable as the cut-off value. Optimal cut-off value of PNI regarding patient prognosis were assessed by means of maximally selected log-rank statistics using the Maxstat package of R software (version 3.2.2., R Foundation for Statistical Computing, Vienna, Austria).^[22]^ Parameters of perioperative treatment with 5 categories were dummy coded to allow independent entry into the final model. All variables in the risk set were assessed as putative prognostic factors for DFS and OS in unadjusted Cox-regression. Variables with a *P* value of less than 0.10 in the unadjusted Cox regression were selected for risk-adjusted Cox-regression. A *P* value of less than 0.05 was considered statistically significant.

## 3 Results

A total of 1258 patients were included in the analysis and their mean follow-up period was 49.5 (±24.2) months. Among these patients, 101 patients developed postoperative AL, giving an AL rate of 8.0%. We compared the two groups of 1157 patients without AL versus 101 patients with AL. The comparisons of the characteristics between 2 groups are summarized in Table 1. Patients with AL were younger than those without AL (*P* = 0.003). There were more male patients and a higher proportion of patients undergoing minimal invasive surgery in AL group compared with the non-AL group (*P* < 0.001, *P* = 0.001). Comparing the pattern of recurrences, there was no significant difference for the incidence of systemic recurrence between two groups. However, the incidence of local recurrence was higher in AL group (11.9% vs. 4.8%, *P* = 0.002). Patients in AL group received more preoperative chemo-radiotherapy (*P* = 0.031) and had a longer interval between surgery and postoperative adjuvant treatment compared with patients without AL (*P* < 0.001). Patients with AL also had a longer hospital stay (*P* < 0.001). There were no significant differences between patients with and without AL in other variables such as ASA grade, preoperative CEA level, pathologic stage, histologic grade, lymphovascular invasion, and postoperative chemo-radiotherapy.

**Table 1:**
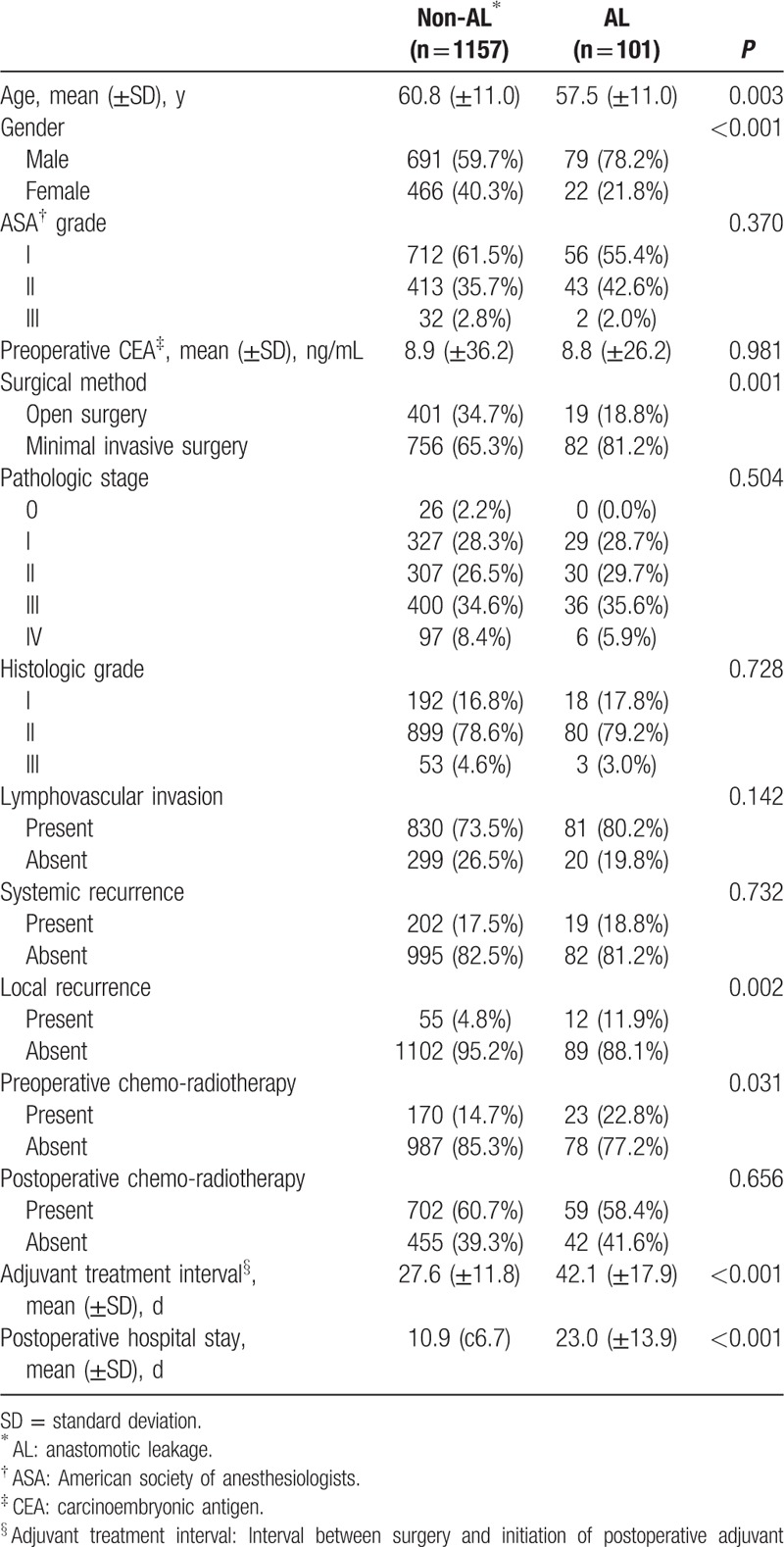
Characteristics of overall patients.

**Figure 1:**
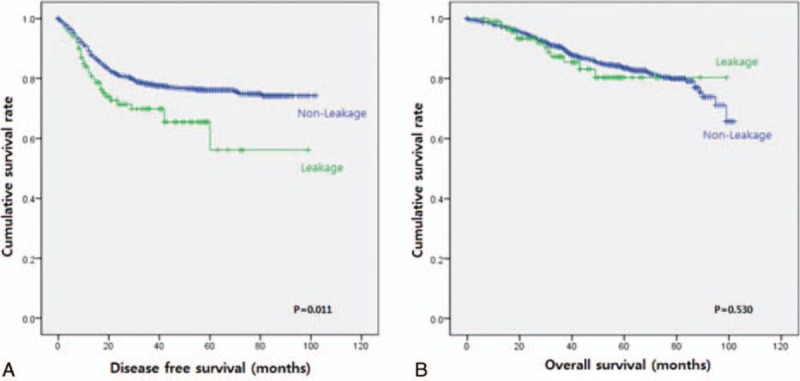
Kaplan–Meier curve for disease free survival and overall survival in patients with (green line) and without (blue line) anastomotic leakage: A. Comparison of disease free survival between (*P* = 0.011). B. Comparison of overall survival (*P* = 0.530).

With regards to DFS, there was a significant difference between the patients with and without AL (*P* = 0.011) (Fig. 1A). Patients with AL had a 3-year DFS of 69.8% and 5-year DFS of 56.1%. Patients without AL had 3-year DFS of 78.0% and 5-year DFS of 76.1%. In OS, there was no significant difference between these two groups (*P* = 0.530) (Fig. 1B). Patients with AL had 3-year and 5-year OS of 87.3% and 80.3%, respectively. Patients without AL had 3-year and 5-year OS of 90.4% and 83.5%, respectively. In the unadjusted Cox-proportional hazards regression, AL was found to be a poor prognostic factor for DFS (hazard ratio [HR] = 1.6; 95% confidence intervals [CI]: 1.1–2.3; *P* = 0.012). Other variables that were associated with poorer DFS included surgical approach, preoperative CEA level, pathologic stage, histologic grade, lymphovascular invasion, and perioperative treatment. After adjusting for potential confounding factors in the risk-adjusted Cox-regression analysis, AL was confirmed to have a statistically significant influence on DFS (HR = 1.6; 95% CI: 1.1–2.4; *P* = 0.012), along with other factors such as elevated preoperative CEA (HR = 1.3; 95% CI: 1.0–1.7; *P* = 0.026), advanced pathologic stage (HR = 2.6; 95% CI: 2.1–3.1; *P* < 0.001) and presence of lymphovascular invasion (HR = 1.6; 95% CI: 1.2–2.0; *P* = 0.001) (Table 2).

**Table 2:**
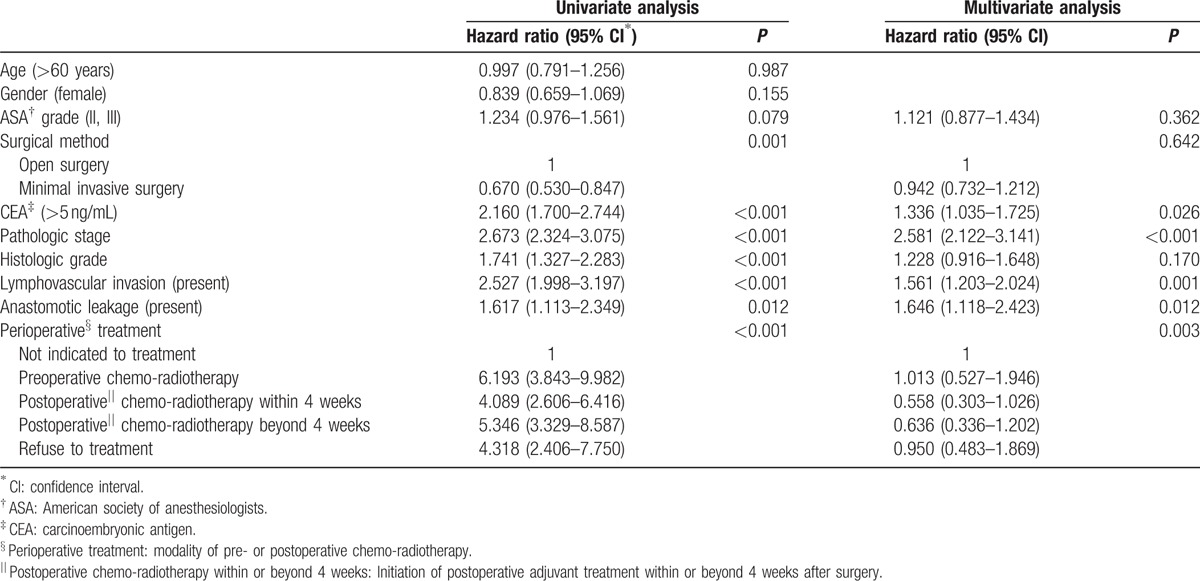
Factors associated with disease-free survival of overall patients.

The indices reflecting clinical severity and degree of inflammation or immunologic reaction are summarized in Table 3. Among the 101 patients with AL, there were 17 (16.8%), 57 (56.4%), and 27 (26.7%) patients with severities of Clavien-Dindo grade 2, 3, and 4, respectively. Thirty-four (36.2%) patients had raised leukocyte count (>11,000/mm^3^) with the other 67 patients (63.8%) not showing biochemical evidence of leukocytosis. Seventy patients (69.3%) showed an increased neutrophil-proportion above normal levels (>80%). For the PNI, the cut-off point in relation to DFS was calculated as 36. There were 29 patients (28.7%) with a PNI above 36. In the unadjusted Cox-proportional hazards regression, Clavien-Dindo grade of AL, neutrophil–proportion, and PNI were not associated with risk of recurrence (HR = 1.0; 95% CI: 0.6–1.7; *P* = 0.992, HR = 1.9; 95% CI: 0.9–3.8; *P* = 0.085, HR = 2.6; 95% CI: 1.0–6.8; *P* = 0.051). Suppressed leukocyte count was associated with increased risk of recurrence (HR = 2.6; 95% CI: 1.0–6.5; *P* = 0.04). However, after adjusting for potential confounding factors in the risk-adjusted Cox-regression analysis, suppressed neutrophil-proportion and decreased PNI were found to have a statistically significant relationship with DFS (HR = 2.6; 95% CI: 1.2–5.8; *P* = 0.02, HR = 3.5; 95% CI: 1.2–9.6; *P* = 0.02), along with the other factors of age over 60 (HR = 2.2; 95% CI: 1.1–4.7; *P* = 0.033) and advanced pathologic stage (HR = 2.4; 95% CI: 1.4–4.0; *P* = 0.001) (Table 4). In the Cox-proportional hazards regression for OS, no biochemical indices were associated with OS. Age over 60 (HR = 6.4; 95% CI: 1.8–23.7; *P* = 0.005) and advanced pathologic stage (HR = 4.1; 95% CI: 1.6–10.7; *P* = 0.004) were associated with poor OS (Table 5).

**Table 3:**
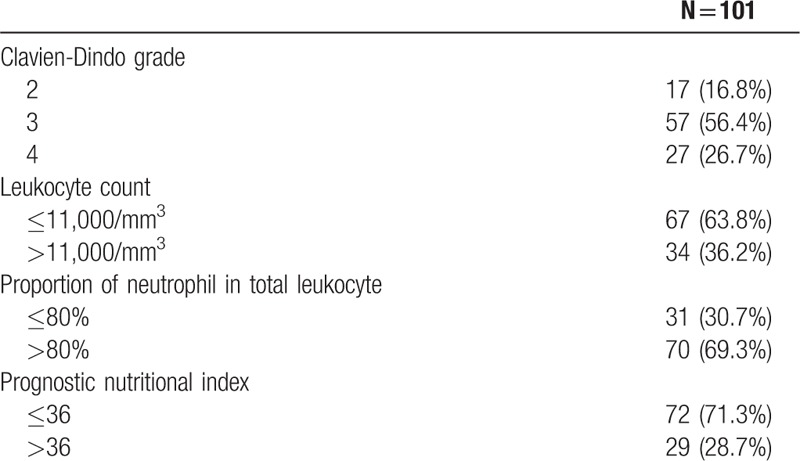
Clinical severity indices of patients with anastomotic leakage.

**Table 4:**
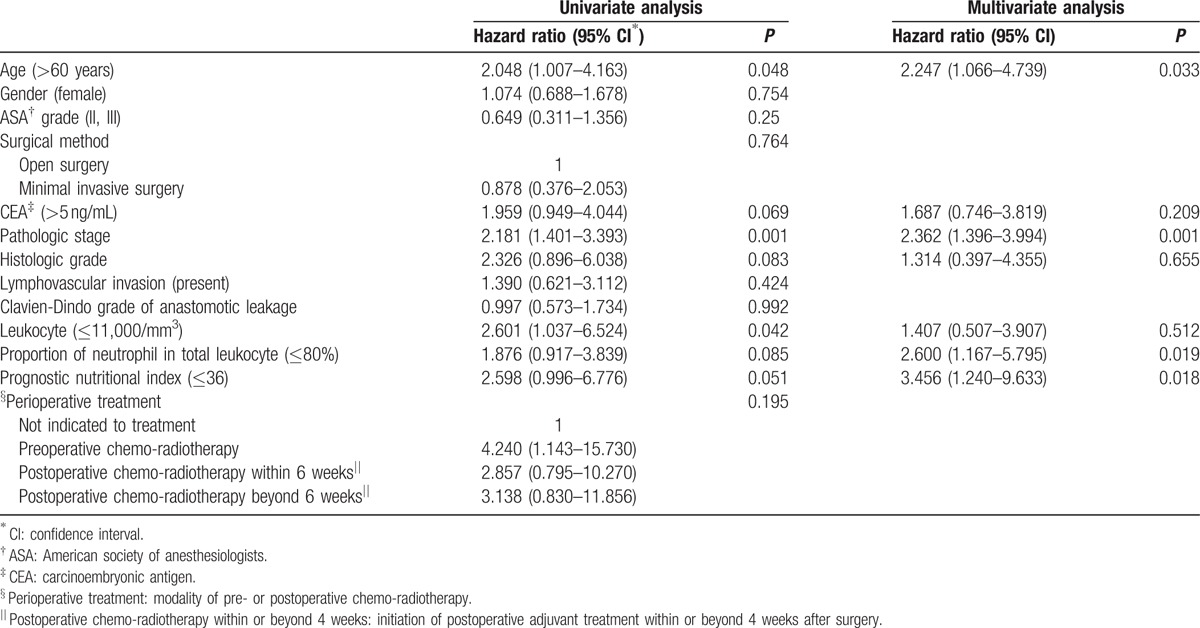
Factors associated with disease-free survival of patients with anastomotic leakage.

**Table 5:**
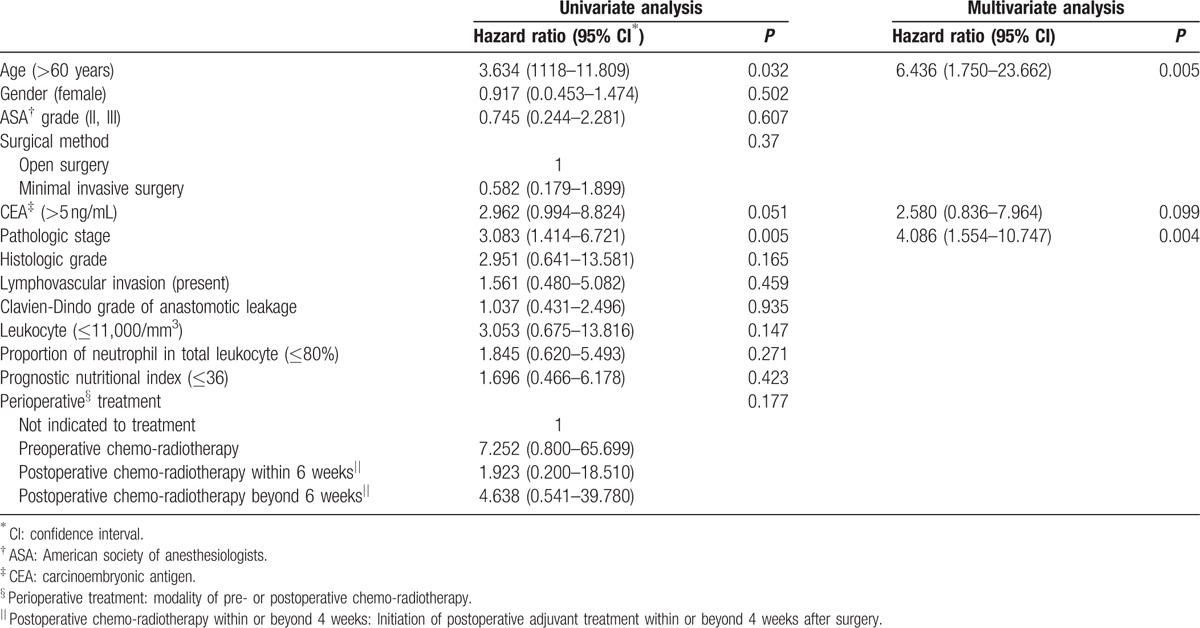
Factors associated with overall survival of patients with anastomotic leakage.

## 4 Discussion

In our study, 8.0% of patients developed AL after LAR without protective stoma, which is similar to the results of previous reports.^[1–6]^ The key finding of this study is that patients with AL show increased risk of tumor recurrence compared with patients without AL. Furthermore, in patients with AL, inflammatory indices such as neutrophil-proportion and PNI are associated with poor oncologic outcomes. Interestingly, there was no significant difference of OS between patients with and without AL. This might be result of adequate post-recurrence management that consisted of curative resection and sequential chemotherapy for resectable recurrence and intensive chemotherapy and/or radiotherapy for unresectalbe recurrence.

Although the pathogenesis of the association between AL and tumor recurrence remains uncertain, several authors have suggested various possible hypotheses: one suggested mechanism is that remnant intraluminal tumor cells can disseminate through the leakage site into the peritoneum, thus increasing tumor recurrence rates.^[23–25]^ In vitro and experimental animal models demonstrate that viable cancer cells detected in the bowel lumen and on suture or staple lines during surgery retain significant growth and metastatic potential.^[23,26–29]^ Inadvertent perforation of the bowel during surgery has been associated with a significantly higher local recurrence rate and reduced overall survival.^[30,31]^ These findings support the notion that AL may lead to extra-luminal implantation of exfoliated cancer cells from the bowel lumen.^[18]^ Another proposed mechanism is that the systemic inflammatory response syndrome caused by AL may play an important role in survival.^[32]^ McArdle et al^[33]^ noted the systemic inflammatory response syndrome may stimulate proliferation and metastasis in residual tumor cells. The release of pro-inflammatory cytokines and growth factors as part of the systemic inflammatory response secondary to intra-abdominal sepsis and associated immune suppression may have a direct effect on the growth of residual tumor cells.^[34–36]^ In our study, we adopted leukocyte count, neutrophil –proportion and PNI as biochemical indices, which were effective and easy-to use serum indicators. Leukocyte count and neutrophil-proportion are traditional indices to estimate the inflammatory reaction. PNI, which is calculated based on the serum albumin concentration and total lymphocyte count, is thought to be a simple and useful parameter to determine the immunological and nutritional status of patients.^[21]^ On multivariate analysis for DFS, suppressed neutrophil-proportion and decreased PNI was associated with increased risk of tumor recurrence. Intra-abdominal sepsis-induced immunosuppression could alter the natural activity of immune system against inflammation. This altered immunity may appear as suppressed neutrophil-proportion and cause incomplete tumor suppression.

Comparing the interval between surgery and postoperative adjuvant treatment as well as the duration of hospital stay after surgery, there were significant differences between patients with and without AL in this study. These results of delays in initiation of adjuvant treatment and in patients’ recovery are frequent events in patients who develop AL. Due to the possibility of these delays influencing oncologic outcome, we included it as a confounding factor along with preoperative chemo-radiotherapy in the multivariate analysis for survival.^[37,38]^

We acknowledge the limitations of the present study. By its retrospective nature, there may be uncontrollable and unrecognized biases: for example, in our analysis, patients without AL were of an older age than patients with AL. This result may be due to the selection of patients for fecal diversion. Patients with old age and poor general health are more likely to have undergone fecal diversion, as this would prevent the potential severe clinical manifestations of AL as well as the consequent requirement for reoperation.^[39,40]^ Also in our study, patients with protective stoma were excluded to clarify the diagnosis of AL and it is the likely reason why the patients with AL were of younger age. Meanwhile, the number of patients with AL was quite low than without AL. This unmatched comparison might have possible risk for bias. However, it was unavoidable in this observational study and over 100 of patients with AL had enough strength for statistical analysis. Also, multivariate analysis for the major end point of oncologic outcome was performed to reduce the risk of bias. Despite these limitations, however, this study has provided unique analyses of details of factors such as the severity of AL, the interval between surgery and postoperative chemo-radiotherapy along with known putative prognostic factors. These comprehensive analyses consider not only the severity of the systemic inflammatory response of AL but also the consequent adverse outcome of delayed postoperative adjuvant treatment which may provide further insight into the treatment of rectal cancer. Certainly, further studies will be required to further advance our knowledge of the oncological consequences of AL to better help with management of such patients.

## 5 Conclusions

AL was associated with poor oncologic outcomes, especially in DFS. In patients with AL, besides the established poor prognostic factors such as age over 60 and advanced pathologic stage, a suppressed neutrophil-proportion as well as a decreased PNI below 36 were associated with tumor recurrence.
